# Effects of glucose and blood pressure control on diabetic kidney disease in old patients with type 2 diabetes

**DOI:** 10.1186/1758-5996-6-81

**Published:** 2014-07-29

**Authors:** Tzu-En Wu, Yu-Hsin Chen, Harn-Shen Chen

**Affiliations:** Division of Endocrinology and Metabolism, Department of Medicine, Taipei Veterans General Hospital, Taipei, 11217 Taiwan; Faculty of Medicine, National Yang-Ming University School of Medicine, Taipei, Taiwan; Division of Nephrology, Department of Medicine, Taipei City Hospital Yangming Branch, Taipei, Taiwan; Department of Ophthalmology, Shin Kong Wu Ho-Su Memorial Hospital, Taipei, Taiwan

**Keywords:** Type 2 diabetes, Old patients, Blood pressure, Diabetic kidney disease

## Abstract

**Aims:**

We determined the influence of age on the effects of glucose and blood pressure control on diabetic kidney disease in patients with type 2 diabetes.

**Methods:**

A total of 721 patients with type 2 diabetes, aged 41–85 years and with an estimated glomerular filtration rate (eGFR) ≥30 mL/ [min · 1.73 m^2^], were enrolled in this study between August 2001 and December 2002. All participants were followed up at our clinics until December 31, 2010. Primary outcomes were the development of end-stage renal disease (ESRD) and all-cause mortality. Secondary outcomes were the development of clinical albuminuria and a severe decline in eGFR.

**Results:**

During the follow-up period (median: 8.3 years), 27 (3.7%) patients developed ESRD, 130 (18.0%) patients died without developing ESRD, and 16 (2.2%) patients died after developing ESRD. Mortality rate increased with age, but the incidence rate of ESRD did not. Poor glucose and blood pressure control was associated with the development of clinical albuminuria and with a severe decline in eGFR in younger patients with diabetes, but not in older patients. The development of severe decline in eGFR and ESRD was significantly lower in the middle tertile of blood pressure (i.e., SBP of 128–141 mm Hg) in older patients.

**Conclusions:**

Adequate glucose and blood pressure control did not reduce the risk of ESRD; however, it may have delayed the onset of clinical albuminuria as well as eGFR decline in younger patients with type 2 diabetes.

**Electronic supplementary material:**

The online version of this article (doi:10.1186/1758-5996-6-81) contains supplementary material, which is available to authorized users.

## Introduction

Diabetic kidney disease is a major cause of morbidity and mortality in people with diabetes, and it is the single leading cause of end-stage renal disease (ESRD) in many countries. Approximately 37.2% of all prevalent ESRD cases and 43.3% of all new ESRD cases in the United States are currently attributed to diabetes [1]. Populations are ageing worldwide, and type 2 diabetes is common among elderly individuals. Some guidelines and review articles have published special recommended considerations for the care of older patients with diabetes [2–6]. Diabetic kidney disease is characterized by the slow development of albuminuria and a progressive decline in glomerular filtration rate (GFR) [7]. However, despite the generally slow progression of kidney disease, mortality rates sometimes significantly exceed ESRD rates, especially in older patients with early stages of kidney disease. According to the UK Prospective Diabetes Study (UKPDS), mortality rates exceeded renal replacement therapy rates among type 2 diabetes patients with clinical albuminuria and elevated plasma creatinine (Cr) levels [8].

Elderly patients with type 2 diabetes are associated with an increased risk of adverse outcomes, including ESRD and death. A number of factors potentially contribute to these outcomes including poor glycemic control, hypertension, dyslipidemia, and smoking [9, 10]. The beneficial effects of diabetes control in reducing the risk of developing ESRD may be confounded by age in older patients with diabetes, due to the competing risks of ESRD and death. Therefore, the primary goal of the present study was to elucidate the beneficial effects of glucose and blood pressure control in older patients with diabetes. We specifically examined the influence of age on advanced diabetic kidney disease in patients with type 2 diabetes whose treatment involved glucose and blood pressure control.

## Subjects and Methods

### Study population

**Figure 1:**
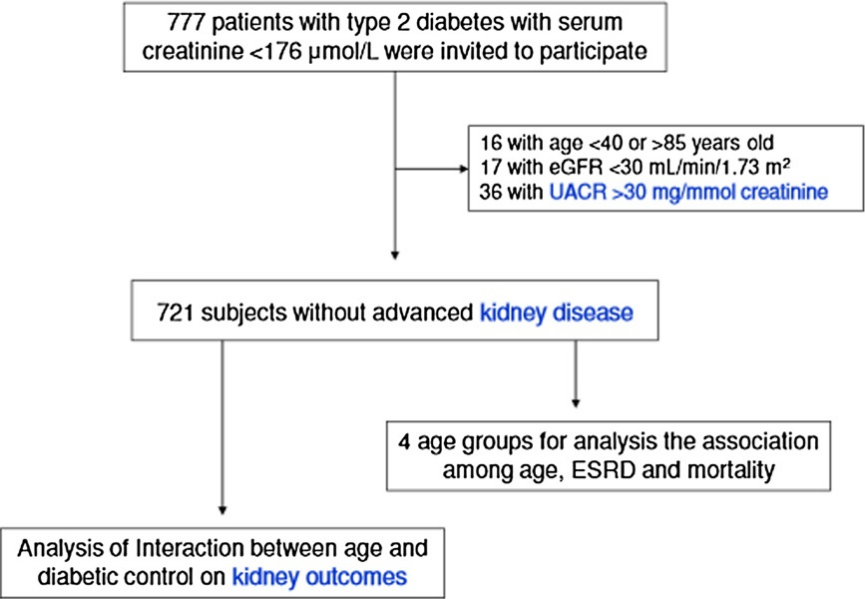
**Flow diagram of the study population.**

A total of 777 patients with type 2 diabetes, who were regularly followed up at the Taipei Veterans General Hospital in Taiwan, initially provided informed consent for participating in this study. Baseline data were collected between August 2001 and December 2002. Serum Cr, fasting blood glucose, glycated hemoglobin (HbA1c), serum cholesterol, triglyceride, urine albumin, and urine Cr levels, as well as blood pressure were subsequently measured for all patients. Patients with cardiovascular disease, malignancy, or serum Cr levels >176 μmol/L, and those who were pregnant were excluded. Patients aged <40 years, aged >85 years, with an estimated glomerular filtration rate (eGFR) <30 mL/[min · 1.73 m^2^], or with clinical albuminuria were also excluded. The remaining 721 patients were treated at our clinics, and were repeatedly examined until December 31, 2010. The flow diagram for the study is presented in the Figure 1. This study was approved by the Institutional Review Board of the Taipei Veterans General Hospital, and written informed consent was obtained from all participants. The reporting of this study conformed to strengthening the reporting of observational studies in epidemiology (STROBE) statement, with references to the STROBE statement [11] and the broader EQUATOR guidelines [12].

### Baseline examination

Blood pressure was measured in a sitting position after 10 min of rest, using an electric blood pressure monitor. Venipuncture was performed, and fasting serum samples were collected for examination. We estimated eGFR using the 4-variable equation from the Modification of Diet and Renal Disease study [13]. Spot urine samples were prepared early in the morning, and the urine albumin-to-creatinine ratio (UACR) was calculated. Duration of diabetes, cigarette smoking, and medication use were also determined for all participants.

### Follow-up examination

All patients were followed up at our clinics until December 31, 2010, according to the national guidelines. In general, HbA1c levels were determined every 3–4 months, while UACR as well as serum Cr, total cholesterol, HDL cholesterol, and triglyceride levels were determined every 6–12 months. Eye fundi examinations were also performed every 6–12 months. Median values for parameters were determined for each year, and mean values for these parameters were determined for the time-weighted average. For example, the median HbA1c value for each year and the mean value for the time-weighted average of HbA1c, presented as mean HbA1c, were determined. Since primary outcomes were ESRD and mortality, the time-weighted average parameters were calculated until the development of ESRD, death, or December 31, 2010.

### Study outcomes

Primary outcomes were the development of ESRD (i.e., initiation of dialysis, renal transplantation, or eGFR <15 mL/ [min · 1.73 m^2^]) and all-cause mortality. Secondary outcomes were the development of clinical albuminuria (UACR >30 mg/mmol creatinine) and a severe decline in eGFR (<30 mL/ [min · 1.73 m^2^]). If no event was reported, observations were censored to the last surveillance time for each patient and outcome. If an event was reported, the event time was recorded as the midpoint between the time of event discovery and the most recent surveillance time. Outcomes data were determined based on the period from baseline to December 31, 2010.

### Assays

HbA1c levels were measured through high-performance liquid chromatography (HPLC), using HLC-723 GHb IIIs (Tosoh, Japan) prior to May 2007 and HLC-723 G7 (Tosoh, Japan) after May 2007. Both urine albumin and Cr levels were assessed using spot urine specimens collected early in the morning. Urinary albumin concentrations were measured by rate nephelometry (IMMAGE® Immunochemistry System; Beckman, CA, USA). Urinary Cr concentrations were measured using a Hitachi 7600 automatic analyzer (Hitachi Ltd., Tokyo, Japan).

### Statistical analysis

Statistical analyses were performed using IBM SPSS Statistics version 19.0 for Windows. Data were expressed as mean ± standard deviation, unless otherwise indicated. UACR and serum triglyceride level were expressed as median values with interquartile ranges (IQR), and analyzed using non-parametric tests. Continuous variables were analyzed via one-way analysis of variance (ANOVA). Non-continuous data were presented as percentages, and were analyzed using Pearson’s χ^2^ test. In order to examine the influence of the interaction, between age on one hand and glycemic and blood pressure control on the other hand, on outcomes, we further divided study participants according to their mean HbA1c and systolic blood pressure (SBP) tertiles. Hazard ratios (HR) and 95% confidence intervals (CI) were calculated using multivariable Cox regression models, after adjusting for other baseline risk factors or potential confounders. For example, in order to compare glycemic control between different age groups, the results were not adjusted for age and HbA1c. Unadjusted incidence rates were calculated through dividing the number of participants with a given complication, by the total number of patient-years including the follow-up period; rates were reported as events per 1000 patient-years. Statistical significance was considered at *P* < 0.05.

## Results

### Clinical characteristics of participants

The mean age of the 721 participants with type 2 diabetes was 67.6 years, and their mean duration of diabetes was 10.1 years. The mean HbA1c level was 7.90% ± 1.66%, mean BMI value was 25.5 ± 3.1 kg/m^2^, mean total cholesterol level was 5.13 ± 0.94 mmol/L, median triglyceride level was 1.91 mmol/L (IQR, 1.06–2.23), mean serum Cr level was 99.9 ± 25.6 μmol/L, mean eGFR was 67.1 ± 17.5 mL/[min · 1.73 m^2^], and median UACR was 2.18 mg/mmol (IQR,0. 92–9.72).

These patients were divided into 4 age groups according to their age at recruitment: 41-60, 61-70, 71-75, and 76-85 years old in order to have similar number of patients in each group. The characteristics of the study population are presented in Table 1. Since this cohort was recruited from Veterans General Hospital, the older patients were predominantly male. The older patients had a lower BMI, lower diastolic blood pressure, longer diabetes duration, higher HDL cholesterol, higher serum Cr, lower eGFR, and used more calcium channel blockers and aspirin.

**Table 1:** **Baseline characteristics according to the age groups in type 2 diabetic patients**

	41-60 years	61-70 years	71-75 years	76-85 years	P value
Number	184	157	194	186	
Age (years)	52.3 ± 5.4	66.7 ± 2.8	72.9 ± 1.4	77.9 ± 2.0	<0.001
Sex (men/women)	85/99	84/73	160/34	167/19	<0.001
Diabetes duration (years)	6.4 ± 5.7	9.4 ± 7.2	11.6 ± 8.6	12.3 ± 8.3	0.001
Current smokers (%)	19.3	16.9	20.4	17.9	0.851
BMI (Kg/m^2^)	26.2 ± 4.2	26.0 ± 3.5	25.4 ± 3.4	24.7 ± 3.1	0.004
Systolic blood pressure (mmHg)	137.2 ± 17.2	141.8 ± 18.7	143.3 ± 19.2	141.5 ± 18.5	0.070
Diastolic blood pressure (mmHg)	82.3 ± 10.3	78.3 ± 11.2	75.2 ± 10.5	72.3 ± 10.6	0.001
Fasting plasma glucose (mg/dL)	163.4 ± 48.4	164.7 ± 44.7	161.0 ± 44.8	164.3 ± 41.3	0.876
HbA1c (%)	8.00 ± 1.81	7.91 ± 1.66	7.88 ± 1.62	7.77 ± 1.53	0.624
Total cholesterol (mg/dL)	204.9 ± 40.2	197.3 ± 38.5	198.4 ± 36.4	191.2 ± 39.0	0.051
High density cholesterol (mg/dL)	34.3 ± 9.2	37.6 ± 5.9	47.6 ± 8.9	45.3 ± 12.3	0.013
Triglyceride (mg/dL), median (interquartile range)	160 (98-233)	145 (96-220)	125 (86-191)	123 (85-194)	0.071
Serum creatinine (mg/dL)	1.03 ± 0.32	1.15 ± 0.46	1.22 ± 0.40	1.30 ± 0.52	0.001
eGFR (mL/min per1.73 m^2^)	75.5 ± 23.1	65.1 ± 17.7	64.6 ± 20.5	60.8 ± 17.2	0.001
Urine albumin-to-creatinine ratio (mg/g), median (interquartile range)	17.5 (7.5-115.7)	18.8 (8.1-113.8)	31.6 (11.0-125.1)	24.6 (12.9-106.7)	0.293
Glucose lowering treatment					
Insulin (%)	35.1	29.3	40.2	26.0	0.150
Sulfonylureas (%)	62.6	69.9	61.5	57.3	0.339
Metformin (%)	73.3	70.7	64.1	57.3	0.069
Thiazolidinediones (%)	13.0	15.4	8.5	8.3	0.383
Other (%)	10.7	14.6	17.1	10.4	0.354
Cardiovascular medication					
ACE inhibitor or ARB (%)	48.1	50.4	59.8	59.3	0.110
Calcium channel blocker (%)	33.6	44.7	51.3	61.5	0.007
Beta-blocker (%)	28.2	26.8	29.9	34.4	0.658
Diuretics (%)	16.0	24.4	33.3	24.6	0.183
Statin (%)	29.8	28.5	21.4	15.6	0.064
Fibrate (%)	2.3	3.1	1.0	2.1	0.143
Aspirin (%)	23.7	32.5	43.6	43.8	0.002

*Abbreviations*: *BMI* Body mass index, *HbA1c* Glycated hemoglobin, *ACE* Angiotensin converting enzyme, *ARB* Angiotensin receptor blocker.

A total of 5002 patient-years were available for examining mortality rates and kidney outcomes during the follow-up period (median: 8.3 years; mean: 6.9 years). Accordingly, 144 (20.0%) patients developed clinical albuminuria, 78 (10.8%) patients had an eGFR <30 mL/ [min · 1.73 m^2^], 27 (3.7%) patients developed ESRD, and 146 (20.2%) patients died. In order to evaluate the effect of the interaction between age and diabetes control on kidney disease, we classified our subjects into 2 age groups: older patients aged 71–85 years, and younger patients aged 41–70 years.

### The influence of age between glucose control and diabetcic kidney disease

Additional file 1: Figure S1 showed the glycemic control and cumulative incidence of outcomes. Table 2 presents the incidence and HR of outcomes, according to the interaction between age and glycemic control. The risk of developing clinical albuminuria was associated with poor glycemic control in younger patients, but not in older patients. Severe decline in eGFR was only associated with poor glycemic control in younger patients with diabetes. Moreover, poor glycemic control did not increase the risk of ESRD and mortality in our study population.

**Table 2:** **Incidence and hazard ratios of nephropathy and mortality according to age and glycemic control**

	Time-weighted average of HbA1c		
	<7.3% (1st tertile)	7.3–8.5% (2nd tertile)	>8.5% (3rd tertile)
**Aged 41–70 years (patient-years)**	102 (734)	113 (773)	126 (843)
Clinical albuminuria			
Events and rate (per 1000 patient-years)	15 (20.4)	19 (24.6)	28 (33.2)
Adjusted hazard ratio	1	1.07 (0.54–2.12)	1.64 (1.07–3.14)
eGFR <30 mL/[min · 1.73 m^2^]			
Events and rate (per 1000 patient-years)	6 (8.17)	7 (9.06)	12 (14.2)
Adjusted hazard ratio	1	1.15 (0.38–5.52)	1.99 (1.07–5.34)
End-stage renal disease			
Events and rate (per 1000 patient-years)	1 (1.36)	6 (7.76)	5 (5.93)
Adjusted hazard ratio	1	5.47 (0.66–45.70)	4.34 (0.49–38.13)
All-cause mortality			
Events and rate (per 1000 patient-years)	11 (15.0)	16 (20.7)	14 (16.6)
Adjusted hazard ratio	1	1.63 (0.75–3.51)	1.61 (0.72–3.61)
**Aged 71–85 years (patient-years)**	139 (940)	127 (829)	114 (741)
Clinical albuminuria			
Events and rate (per 1000 patient-years)	24 (25.5)	28 (33.8)	30 (40.5)
Adjusted hazard ratio	1	1.10 (0.64–1.90)	1.46 (0.85–2.51)
eGFR <30 mL/[min · 1.73 m^2^]			
Events and rate (per 1000 patient-years)	19 (20.2)	16 (19.3)	18 (24.3)
Adjusted hazard ratio	1	0.95 (0.50–1.81)	1.00 (0.51–1.94)
End-stage renal disease			
Events and rate (per 1000 patient-years)	7 (7.45)	3 (3.62)	5 (6.75)
Adjusted hazard ratio	1	0.44 (0.11–2.75)	0.87 (0.28–2.75)
All-cause mortality			
Events and rate (per 1000 patient-years)	44 (46.8)	27 (32.6)	34 (45.9)
Adjusted hazard ratio	1	0.68 (0.42–1.10)	1.04 (0.66–1.63)

*Abbreviations*: *HbA1c* Glycated hemoglobin, *eGFR* Estimated glomerular filtration rate.

Adjusted for sex, duration of diabetes, body mass index, smoking status, blood pressure, total cholesterol, HDL cholesterol, serum creatinine, urine albumin-to-creatinine ratio, and medications.

### The influence of age between blood pressure control and diabetic kidney disease

Additional file 2: Figure S2 showed the blood pressure control and cumulative incidence of outcomes. Table 3 presents the incidence and HR of outcomes, according to the interaction between age and blood pressure control. The risk of developing clinical albuminuria was associated with high blood pressure in younger patients, but not in older patients. A severe decline in eGFR was only associated with high blood pressure in younger patients with diabetes, and high blood pressure caused a significant increase in mortality among younger patients. The HR of advanced kidney disease, including severe decline in eGFR and the development of ESRD, was significantly lower in the middle tertile of blood pressure (i.e., SBP of 128–141 mm Hg) in older patients.

**Table 3:** **Incidence and hazard ratio of nephropathy and mortality according to age and blood pressure control**

	Time-weighted average of SBP (mm Hg)		
	<128 (1 ^st^ tertile)	128-141 (2 ^nd^ tertile)	>141 (3 ^rd^ tertile)
**Aged 41–70 years (patient-years)**	129 (938)	102 (712)	110 (764)
Clinical albuminuria			
Events and rate (per 1000 patient-years)	12 (12.8)	17 (23.9)	33 (86.4)
Adjusted hazard ratio	1	1.90 (0.91–3.98)	3.70 (1.91–7.17)
eGFR <30 mL/[min · 1.73 m^2^]			
Events and rate (per 1000 patient-years)	5 (5.33)	9 (12.6)	11 (14.4)
Adjusted hazard ratio	1	2.44 (0.81–7.29)	2.67 (1.03–7.67)
End-stage renal disease			
Events and rate (per 1000 patient-years)	2 (2.13)	4 (5.62)	6 (7.85)
Adjusted hazard ratio	1	2.71 (0.49–14.88)	3.56 (0.72–17.67)
All-cause mortality			
Events and rate (per 1000 patient-years)	13 (13.9)	10 (14.0)	18 (23.6)
Adjusted hazard ratio	1	1.04 (0.46–2.38)	1.77 (1.07–3.61)
**Aged 71–85 years (patient-years)**	120 (816)	136 (911)	124 (860)
Clinical albuminuria			
Events and rate (per 1000 patient-years)	25 (30.6)	33 (36.2)	24 (27.9)
Adjusted hazard ratio	1	1.09 (0.64–1.84)	1.03 (0.59–1.81)
eGFR <30 mL/[min · 1.73 m^2^]			
Events and rate (per 1000 patient-years)	26 (31.9)	13 (14.3)	14 (16.3)
Adjusted hazard ratio	1	0.45 (0.23–0.89)	0.62 (0.32–1.20)
End-stage renal disease			
Events and rate (per 1000 patient-years)	10 (12.3)	2 (2.20)	3 (3.49)
Adjusted hazard ratio	1	0.21 (0.04–0.95)	0.36 (0.10–1.32)
All-cause mortality			
Events and rate (per 1000 patient-years)	43 (52.9)	33 (36.2)	29 (33.7)
Adjusted hazard ratio	1	0.68 (0.43–1.09)	0.64 (0.40–1.03)

*Abbreviations*: *SBP* Systolic blood pressure, *eGFR* Estimated glomerular filtration rate.

Adjusted for sex, duration of diabetes, body mass index, smoking status, HbA1c, total cholesterol, HDL cholesterol, serum creatinine, urine albumin-to-creatinine ratio, and medications.

### Associations among age, ESRD, and mortality

During the study period, 27 patients developed ESRD (3.7%, 5.4 per 1000 patient-years), 16 patients died after developing ESRD (2.2%, 3.2 per 1000 patient-years), 130 patients died without developing ESRD (18.0%, 26.0 per 1000 patient-years), and 564 patients were alive and had not developed ESRD (78.2%).

**Figure 2:**
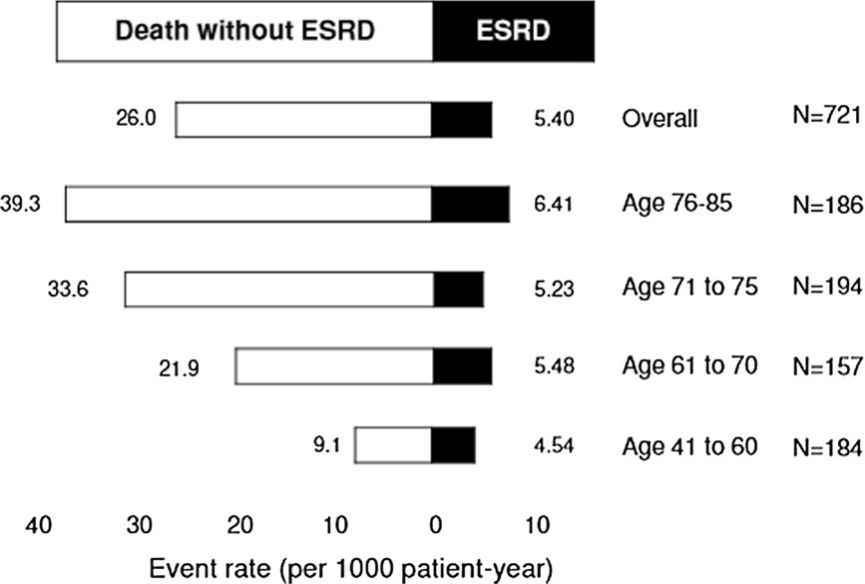
**Incidence of ESRD and pre-ESRD mortality during the follow-up period according to age group.** There were 27 patients who developed ESRD (3.7%, 7.51 per 1000 patient-years), and 130 patients who died without developing ESRD (18.0%, 28.3 per 1000 patient-years). The incident rate of ESRD was not significantly different between age groups, but the mortality rate increased with age.

Incidence of ESRD and of pre-ESRD mortality stratified by age is presented in Figure 2. The patients with age between 41 and 60 years were used for reference to assess the effects of age on ESRD and mortality (Figure 2). The unadjusted hazard ratio (HR) for mortality was 2.74 (95% CI, 1.42–5.27) for patients aged between 61 and 70 years, 4.04 (95% CI, 2.20–7.41) for patients aged between 71 and 75 years, and 5.04 (95% CI, 2.76–9.19) for patients aged between 76 and 85 years. The unadjusted HR for ESRD was 1.20 (95% CI, 0.39–3.72) for patients aged between 61 and 70 years, 1.17 (95% CI, 0.39–3.49) for patients aged between 71 and 75 years, and 1.54 (95% CI, 0.54–4.45) for patients aged between 76 and 85 years. Mortality rate increased with age, but the incidence rate of ESRD was not significantly different between age groups.

## Discussion

Our follow-up study, involving a cohort of patients with type 2 diabetes, highlights several interesting findings. During the median follow-up period of 8.3 years, 27 (3.7%) patients developed ESRD, 130 (18.0%) patients died without developing ESRD, and 16 (2.2%) patients died after developing ESRD. Mortality rate significantly increased with age; however, incidence of ESRD was not affected by age. Poor glucose and blood pressure control were associated with the development of clinical albuminuria, but not with severe decline in eGFR or with the development of ESRD. In older patients, however, the HR of a severe decline in eGFR and developing ESRD was significantly lower for the middle tertile of blood pressure (i.e., SBP of 128–141 mm Hg).

Forsblom et al. followed up 592 patients with type 1 diabetes with clinical albuminuria for a median period of 9.9 years, and reported that 56 (9.5%) patients died without developing ESRD, while 210 (35.5%) patients developed ESRD [14]. Moreover, Rosolowsky et al. followed up 423 Caucasian patients with type 1 diabetes who presented with clinical albuminuria for 15 years, and reported that 29 patients died without developing ESRD, while 172 patients developed ESRD [15]. During the entire 5-year follow-up period of the Action to Control Cardiovascular Risk in Diabetes (ACCORD) trial, the mortality rate was 7.5% in the intensive therapy group, compared to 6.5% in the standard therapy group (HR: 1.19; *P* = 0.02) [16]. Simultaneously, the cumulative incidence of ESRD was 2.7% in the intensive therapy group, compared to 3.0% in the standard therapy group (HR: 0.92; *P* = 0.488) [17]. In previous studies of patients with type 1 diabetes, the mean ages of the 2 cohorts were 42 and 34 years, respectively [14, 15]. Participants in the ACCORD trial had a median age of 62 years [16, 17]. In our present study, the mean age of participants was 67.6 years. Therefore, our findings, as well as previously reported results, suggest that older patients with diabetes without advanced kidney disease are at a higher risk of death than developing ESRD.

Several previous studies have indicated that intensive glycemic control may reduce albuminuria; however, results were inconsistent with respect to GFR decline [7]. Our data revealed that younger patients with relatively good glucose and blood pressure control (i.e., mean HbA1c <7.3% and mean SBP <128 mm Hg) had a lower risk of clinical albuminuria and lower GFR than younger patients with poor glycemic control (i.e., mean HbA1c >8.5% and mean SBP >141 mm Hg). However, relatively good glucose and blood pressure control does not seem to reduce the risk of kidney disease in older patients. Patients with diabetic nephropathy are known to be at a higher risk of kidney failure and death. Therefore, the competing risk of death may confound the estimates of the effect of diabetes control on kidney failure. Older patients with diabetes (aged 76–85 years) reportedly have a 5-fold increased risk of death (HR: 5.04; 95% CI, 2.76–9.19) than younger patients with diabetes (aged 41–60 years). When the life expectancy of this cohort was estimated using the UKPDS Outcomes Model [18], the life expectancy of the younger and older patients was determined to be 19.2 years (IQR, 17.7–20.7) and 8.2 years (IQR, 6.5–10.0), respectively. Since the development of ESRD requires time, the findings described above may be due to the limited life expectancy of older patients, and the competing risks of death and ESRD. Thus, the influence of survival on diabetic complications may be stronger than that of diabetes control, such as blood glucose and blood pressure control.

Table 1 reported that the diastolic blood pressure demonstrated a statistical difference between the different age groups in the ANOVA analysis. According to Framingham Heart study, there is a gradual increase in blood pressure with aging and this trend with a larger increase systolic blood pressure than diastolic blood pressure [19]. The systolic blood pressure continues to rise until the ninth decade, but diastolic blood pressure tends to decrease or remain the same after the fifth decade. All of these patients were treated according to our national guideline with the same target of blood pressure. Because the diastolic BP has no significant change with increasing age, it is reasonable that diastolic BP was lower in patients with old age. This is the reason why we chose systolic BP for analysis. The original intension of this study is to investigate the optimal blood pressure and blood glucose control in old patients with diabetes. According to our data, a lower development of severe decline in eGFR and ESRD was in the middle tertile of blood pressure (SBP 128-141 mmHg) in older patients. The Hazard ratio was lower, but not statistically significant, in the highest tertile of blood pressure than in the lowest tertile of blood pressure. These data may be due to the "U" curve (middle is better) for blood pressure. We suggested that in type 2 diabetic patients with age older than 70 years old, the optimal systolic blood pressure may be between 128 and 141 mmHg.

One strong aspect of our study was that it included a significant number of mortality events, even though a low number of ESRD cases was reported. During our follow-up period, Taiwan had the highest incidence and prevalence of ESRD in the world [20]. However, despite the extremely high ESRD rate, mortality rate still exceeded it. Therefore, we were able to provide very powerful evidence of the competing risks of mortality and ESRD in type 2 diabetes. Our study also had some limitations that should be addressed. First, this was an observational study and not a randomized, controlled trial. However, a randomized, controlled trial may be difficult to conduct among older patients with diabetes. Second, due to the generally slow rate of progression of kidney disease, complications such as ESRD usually require a long period of time to develop.

In conclusion, the mortality rate far exceeded the rate of ESRD in patients with type 2 diabetes without advanced kidney disease, especially among older subjects. The high mortality rate may interfere with the evaluation of the favorable effects of diabetes control on diabetic kidney disease. Improving glucose and blood pressure control may have some beneficial outcomes on the development of kidney disease in younger patients, but not in older patients. Our findings suggest that certain surrogate markers such as clinical albuminuria and a severe decline in eGFR may be more appropriate than ESRD for evaluating diabetes control.

## Electronic supplementary material

Additional file 1: Figure S1: Glycemic control and cumulative incidence of outcomes (A, B) clinical albuminuria, (C, D) eGFR <30 mL/[min · 1.73 m^2^], (E, F) ESRD, (G, H) all-cause mortality of the 2 groups of subjects. (TIFF 55 KB)

Additional file 2: Figure S2: Blood pressure control and cumulative incidence of outcomes (A, B) clinical albuminuria, (C, D) eGFR <30 mL/[min · 1.73 m^2^], (E, F) ESRD, (G, H) all-cause mortality of the 2 groups of subjects. (TIFF 62 KB)
