# Supplementary figures and images for: Effects of glucose and blood pressure control on diabetic kidney disease in old patients with type 2 diabetes
Source: Diabetol Metab Syndr. 2014 Jul 29;6:81. doi: 10.1186/1758-5996-6-81 (PMC4124170; doi:10.1186/1758-5996-6-81)

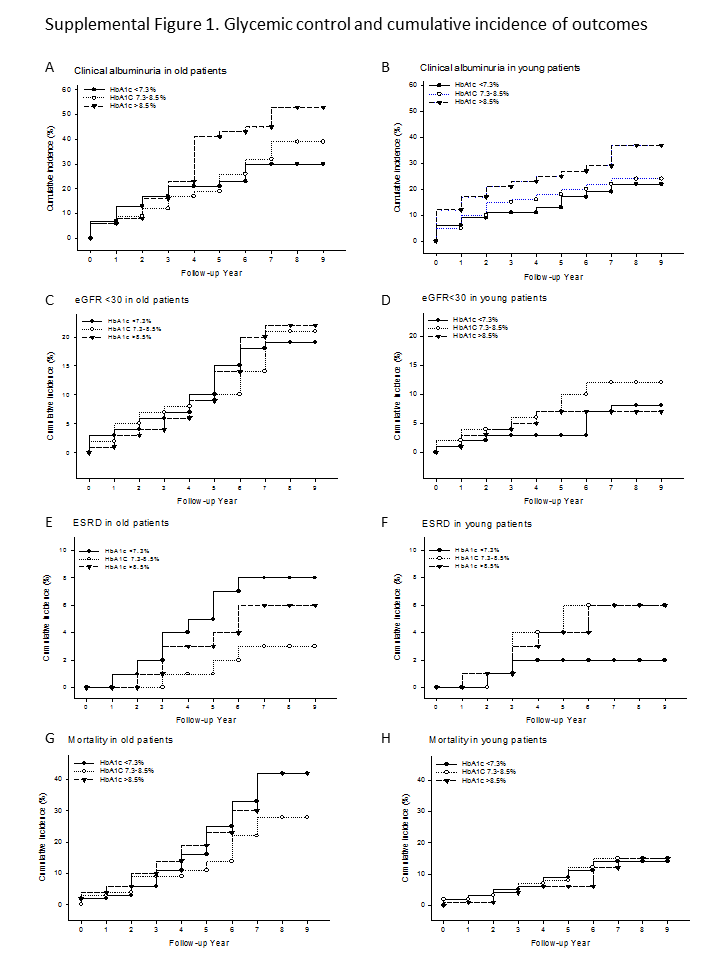

Supplement: Supplementary file 1 — Additional file 1: Figure S1: Glycemic control and cumulative incidence of outcomes (A, B) clinical albuminuria, (C, D) eGFR <30 mL/[min · 1.73 m2], (E, F) ESRD, (G, H) all-cause mortality of the 2 groups of subjects. (TIFF 55 KB) [file 13098_2014_344_MOESM1_ESM.tiff]

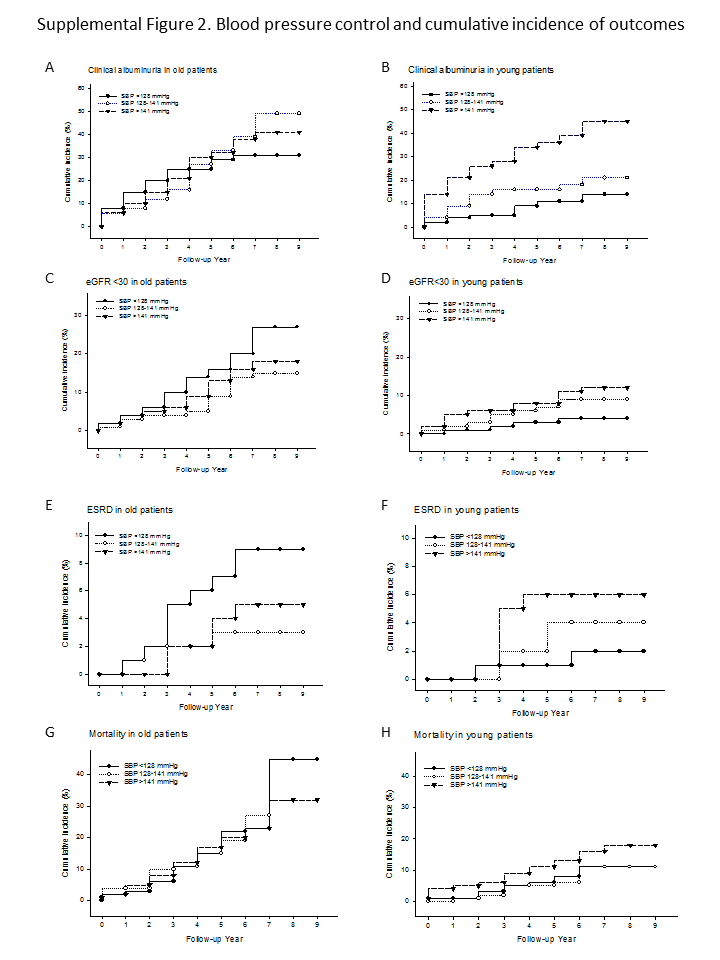

Supplement: Supplementary file 2 — Additional file 2: Figure S2: Blood pressure control and cumulative incidence of outcomes (A, B) clinical albuminuria, (C, D) eGFR <30 mL/[min · 1.73 m2], (E, F) ESRD, (G, H) all-cause mortality of the 2 groups of subjects. (TIFF 62 KB) [file 13098_2014_344_MOESM2_ESM.tiff]
